# Fifteen Years of the Genome Analysis Toolkit as the De Facto Standard in Short-Read Variant Calling

**DOI:** 10.3390/ijms27093754

**Published:** 2026-04-23

**Authors:** Asta Blazyte, Long Le, Jaesuk Lee, Delger Bayarsaikhan, Bonghee Lee

**Affiliations:** 1 School of Medicine, Gachon University, Incheon 21565, Republic of Korea; 2 Vinmec Research Institute of Stem Cells and Gene Technology, Hanoi 100000, Vietnam; 3 nSAGE, Incheon 21999, Republic of Korea

**Keywords:** genome analysis toolkit, GATK, variant calling, DNA, NGS

## Abstract

Genome Analysis Toolkit (GATK) is a rigorously maintained collection of 430 analysis tools and a core bioinformatics engine. First released in 2010 as a toolkit for next-generation sequencing (NGS) data analysis, GATK remains one of the least celebrated yet foundational tools of the NGS era. By employing state-of-the-art approaches and continuously adapting to the evolving demands of NGS analysis, it has effectively unified the variant calling process worldwide. In a field as rapidly evolving as genomics, it is remarkable that, over a decade later, the same toolkit remains the gold standard. This critical review explores the pre-history of GATK, the reasons for its broad and enduring adoption by the scientific community, its developmental evolution, contributions to science, and future prospects.

## 1. Introduction

Next-generation sequencing (NGS) was introduced in the early 2000s [1], providing a fast and low-cost sequencing technology that rather rapidly outcompeted Sanger’s chain-termination method [2]. Before revolutionizing molecular biology and genomics, NGS faced several challenges that needed to be overcome [2]. NGS leveraged the availability of reference genomes, which had been produced by earlier sequencing methods [3], but the DNA fragments generated were sometimes as short as 25 bp [1]. While parallelization reduced sequencing time [2], such short reads were difficult to assemble accurately into long, contiguous sequences [4]. While early short-read technologies made de novo assembly challenging, the availability of high-quality reference genomes shifted many applications toward reference-based analyses [5], thereby placing accurate read mapping and variant detection at the core of NGS pipelines [6].

Aligners were (and remain) essential for mapping NGS reads to reference genomes, playing a key role in the accuracy of variant calling [7]. For a long time, several widely used aligners have dominated the field, including Minimap2 [8], Burrows–Wheeler Aligner (BWA) [9], and Bowtie [10], with specialized tools such as HISAT2 [11], STAR [12], and NovoAlign [13] serving niche purposes such as RNA-seq or high-accuracy DNA mapping. Despite the impressive diversity of alignment tools and their employed state-of-the-art algorithms, this review will focus on the next step: variant calling.

The fundamental principle of reference-guided variant calling is straightforward: a detection algorithm scans and compares aligned reads to a reference genome, identifying base matches and mismatches at each genomic position to infer the genotype [5,14]. However, despite its perceived simplicity, variant calling is an error-prone process sensitive to factors such as read quality, sequencing depth, and the choice of reference genome [5]. The algorithm must navigate the balance between sensitivity and specificity to determine whether an observed variation is a technical artifact or a true genomic variant [5,15]. Early variant callers fell short in several important areas, including the accuracy of indel recognition, systematic error correction, and the ability to correctly determine genotypes at low sequencing depth [5,15,16,17].

In the late 2000s, NGS was already widely used for genomics analyses; however, variant calling remained a tedious process, requiring advanced programming and bioinformatics pipelining skills along with deep knowledge of the experimental and computational artifacts introduced by earlier processing steps [4]. With several major large-scale genomics projects such as the 1000 Genomes Project [18] (launched in 2008 [19]) underway [18,20], the need to establish bioinformatics pipelines and quality standards became evident. In this context, the Genome Analysis Toolkit (GATK) first appeared in 2010 [20], quickly becoming the standard solution, and ultimately the de facto standard, for variant calling [20,21].

**Figure 1:**
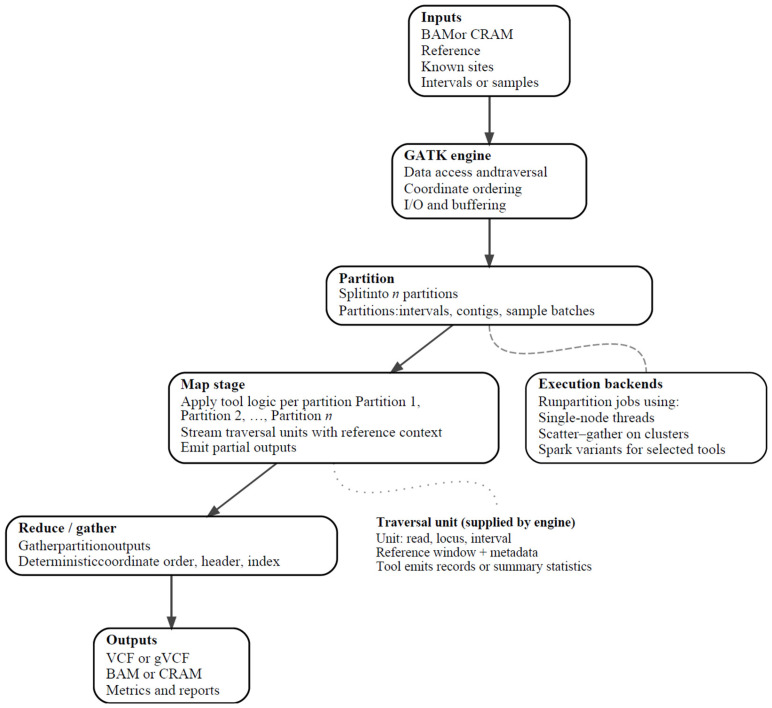
Conceptual overview of the GATK core engine execution model. Solid arrows indicate the main data flow through the engine. Dotted arrows indicate contextual or supporting relationships that influence execution but are not part of the main data processing path. Rounded boxes represent processing stages or infrastructure components, and unboxed text denotes runtime concepts handled by the engine. The figure shows how the GATK engine partitions genomic data into multiple intervals, applies tool logic independently during the *map* stage, and then gathers partial outputs into ordered final result files.

At first glance, the widespread adoption of the GATK by the scientific community might appear to stem primarily from its open-access nature and its origin at one of the earliest bioinformatics hubs in the world, the Broad Institute (Harvard and MIT, Cambridge, MA, USA). However, GATK’s success was rooted in technical innovation. The toolkit was implemented as a platform-independent Java programming framework that employed a MapReduce approach, enabling both shared memory and distributed parallelization of computational tasks at high scalability [20] (Figure 1).

By the time GATK was developed, the MapReduce framework had already been widely implemented by Yahoo! (Sunnyvale, CA, USA) [20] and used by Google (Mountain View, CA, USA) to generate data for its web search [22]. When GATK was introduced in the peer-reviewed journal Genome Research [20], it had already been employed in major large-scale projects such as the 1000 Genomes Project [23,24] (http://www.1000genomes.org) and The Cancer Genome Atlas (http://cancergenome.nih.gov) [25], establishing its credibility [20]. The combination of a robust architecture and early adoption allowed GATK to become a unifying standard for all types of variant calling (e.g., germline and somatic), enabling research groups worldwide to adopt consistent best practices rather than building custom variant calling and filtering pipelines from scratch.

GATK today (version 4.6.1.0 at the time of writing [26]) is a massive open-source toolset managed by the Broad Institute, enabling easy construction of a customized NGS analysis pipeline for almost any genomic data [22,27,28]. The toolset contains 430 tools organized into 21 function-based categories, including short variant discovery, variant filtering, base calling, and variant annotation [28]. GATK can handle a wide spectrum of genomic data, from DNA and RNA to more specialized applications, such as the recently introduced MethylationTypeCaller (https://github.com/broadinstitute/gatk/blob/master/src/main/java/org/broadinstitute/hellbender/tools/walkers/MethylationTypeCaller.java#L31; accessed on 30 March 2026) [21,28].

This review explores the journey of a single genome analysis toolkit that has dominated variant calling for over a decade, with a particular focus on GATK’s technological evolution, scientific contributions, and future prospects.

## 2. GATK Core Engine and Architectural Framework

The GATK core engine is designed as a Java-based data management system, in which the engine coordinates the logistics of data processing from input (*scatter*) to output (*gather*), while individual tools operate only on the subsets of data relevant to their specific analytical task [20]. More specifically, the core engine is traversal-based: it iteratively partitions large NGS inputs, such as the reference genome, sequencing reads, or alignment files, into smaller units and interval partitions (*scatter*), and applies user-specified analyses to each unit in turn [20] (Figure 1). In this framework, the core engine handles data access and traversal order, loading defined genomic intervals together with their associated metadata. Notably, the framework combines per-thread *reduce* outputs using the tree-reduction strategy, which aligns with *scatter-gather* parallelization over genomic intervals [20,29]. Within this architecture, GATK tools operate over engine-defined traversals, allowing analytical logic to remain independent of data iteration and access mechanisms (Figure 1).

To support efficient handling of genomic data, the GATK core engine implements caching and buffering that optimize input–output throughput and memory usage on available computational infrastructure [20]. Metadata required for downstream analyses, including base quality scores and read group information, are standardized by the core engine and passed to the relevant analysis tools in a consistent manner [16]. By separating common data-handling from tool-specific analytical logic, GATK enables robust execution, improved memory management, and increased overall stability across diverse analyses [20] (Figure 1).

In the initial GATK release, the core engine and tools supported four primary traversal types with distinct computational characteristics. These included site-by-site traversal across genomic loci (TraverseLoci), whole-read traversal (TraverseReads), window-based interval traversal (TraverseLocusWindows), and traversal modes designed to operate on duplicate reads (TraverseDuplicates) [20]. Importantly, the modular design of the core engine allowed the introduction of additional traversal types. This separation between the core engine and individual tools also makes the toolkit easier to expand and allows each analysis to use a computation model suited to its specific needs [20].

## 3. GATK’s Evolution and Version History

**Figure 2:**
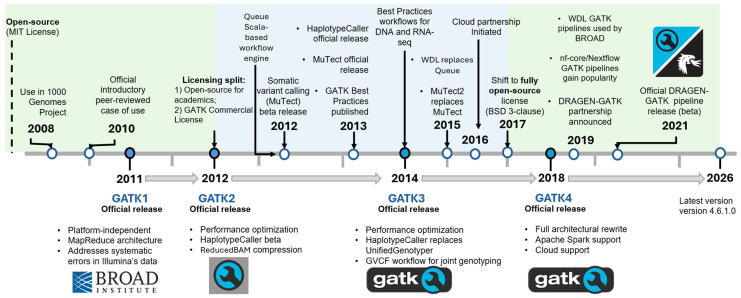
GATK’s evolution and key milestones. Major releases are indicated by blue circles with a black outline, with their major features summarized below. Development periods are shown as horizontal gray arrows. Technical, licensing, and ecosystem transitions are denoted by white circles with blue outlines. Background shading indicates licensing periods: green, fully open-source; blue, open-source/GATK Commercial License.

To date, GATK has evolved through four major versions (Figure 2), featuring one major architectural rewrite of its core engine. These four major GATK releases came with multiple updates each, with some versions of the same release separated by as much as 2–6 years of active development [21] (Table 1). The changes between the major versions reflect both advances in sequencing technologies and evolving requirements for large-scale variant discovery. On the other hand, minor versions of the same release added novel tools to support new NGS data types, and continuously addressed variant calling discrepancies and refined the internal variant calling algorithms used by existing tools, with respect to variant detection accuracy and processing speed [21,30,31]. Throughout four major versions and fifteen years of active development, GATK has addressed numerous sources of artifacts, ranging from experimental errors such as PCR amplification biases, sequencing errors, and platform-specific systematic effects, to computational issues, including software errors and mapping artifacts, automating many of these sophisticated processes [7,14].

The first major stable release (GATK1) was developed in response to limitations of early pileup-based variant callers. Typically, pileup approaches were largely limited to single-nucleotide polymorphism (SNP) discovery and lacked sophistication in their error modeling [20]. GATK1 introduced systematic handling of base quality scores, mapping quality, and probabilistic modeling of platform-specific error patterns [16]. The toolkit included locus-based tools for SNP and small indel discovery and genotyping, such as the AlleleCaller application, which later formed the basis of the UnifiedGenotyper. In addition, GATK1 introduced Base Quality Score Recalibration (BQSR) [16,20,32] (Table 1 and Figure 2), a notable novelty that explicitly modeled and corrected systematic sequencing errors, improving data reliability and downstream variant-calling accuracy [20].

GATK2 was largely an extension of GATK1 that expanded the toolkit and optimized variant discovery workflows [7,14]. During this phase, major advances were made in strict error detection and removal, marked by the introduction of local realignment around indels [7,14,16]. This type of realignment reduced false-positive variant calls caused by alignment artifacts at the indel breakpoints that were previously misinterpreted as clusters of SNPs [16]. Consequently, UnifiedGenotyper extended variant detection beyond SNPs to include short indels while incorporating genotype likelihood models that accounted for sequencing depth, base quality, and mapping uncertainty [14,16]. Moreover, GATK2 included enhancements to multiple core tools, such as UnifiedGenotyper, which gained support for variant calling in pooled samples and variable ploidy, including mitochondrial DNA, and BQSR, which generated a more detailed error model [31,32]. Among newly introduced tools, HaplotypeCaller, a local de novo assembly-based variant caller, was introduced as an experimental beta feature, along with ReduceReads, a BAM compression algorithm able to reduce alignment file size up to 100-fold (Table S1), and MuTect (Table 1 and Figure 2), the first GATK tool dedicated to somatic variant calling, also introduced as an experimental beta feature [31,33].

Since GATK3, HaplotypeCaller largely replaced the older and less accurate UnifiedGenotyper for SNP and indel discovery [14] (Figure 2). This methodological shift improved indel calling accuracy and enabled better representation of complex variants by resolving local haplotypes rather than relying on single-site evidence [30]. GATK3 also emphasized joint genotyping across cohorts through the introduction of the GVCF workflow, enabling scalable multi-sample handling [34] (Table 1). This workflow was designed for large-scale genomic studies, allowing consistent estimation of genotype likelihoods across samples and thereby mitigating batch-related variability [14]. In addition, GATK3 saw improvements in somatic variant detection [35].

With GATK4, the underlying engine architecture was fundamentally rewritten to improve computational efficiency (Figure 2 and Table 1). A key optimization introduced during this release was support for in-memory distributed processing via Apache Spark (https://spark.apache.org/; accessed on 30 March 2026), aiming to resolve the computational skew in some of its core variant calling tools, such as HaplotypeCaller [36]. This change enabled dramatic performance improvements, enhanced scalability across cloud and cluster environments, and more efficient pipelines while retaining variant calling accuracy and Broad tool support [36,37]. For instance, in a workstation benchmarking of Spark-enabled tools in a targeted sequencing workflow, HaplotypeCallerSpark and BQSRPipelineSpark were reported to achieve a combined ~86% reduction in execution time relative to their non-Spark counterparts, while duplicate marking was reduced by ~42%, with an overall concordance of 98% between Spark and non-Spark variant calls [37] (Table S1).

BQSR optimization, on its own, was a notable improvement presented by the GATK team in the ‘2020 Annual IEEE Symposium on Field-Programmable Custom Computing Machines (FCCM)’ and included both an optimized algorithm and hardware-based acceleration [27]. Moreover, GATK4 further expanded support for somatic variant calling through Mutect2 (Figure 2), improved RNA-seq handling, and variant calling of non-diploid genomes [30]. This GATK version also supports copy number variation (CNV) calling via the GermlineCNVCaller tool (https://github.com/broadinstitute/gatk/blob/master/src/main/java/org/broadinstitute/hellbender/tools/copynumber/GermlineCNVCaller.java; accessed on 30 March 2026) [21] (Table 1). As of 2026, GATK4 remains under active development.

**Table 1:** Summary of GATK versions and major improvements.

GATK Version	Release/ Maintenance	Major Architectural/Performance Changes	Sources
**GATK1** v.1.0– v.1.6	2011–2012 7 versions/ patches	Initial release platform-independent Java engine MapReduce architecture introduced Introduced multi-threading Early tools for variant discovery (SNPs, indels) built on top of the framework	[16,20,32]
**GATK2** v.2.0– v.2.8	2012–2013 9 versions/ patches	Early refinements Optimized performance (BQSR, UnifiedGenotyper) Introduction of HaplotypeCaller (beta) ReduceReads BAM compression algorithm Improved algorithm for homopolymer runs Improved multi-threading	[31,32]
**GATK3** v.3.0– v.3.8-1	2014–2018 10 versions/ patches	Mature implementation Emphasis on Best Practices workflows (e.g., joint genotyping, HaplotypeCaller) Performance enhancements: multi-threading, I/O optimization, vector/Pair-HMM optimization ~29% runtime reduction in GATK 3.8 versus unoptimized versions Best Practices for variant calling on RNA-seq data	[32,34,38,39]
**GATK4** v.4.0.0.0– v.4.6.1.0	2018–2025 ~46 versions/ patches	Full architectural rewrite Apache Spark engine for distributed/in-memory processing Up to 15× faster performance and 5× larger input capacity than GATK3 Adopted BSD open-source license Cloud-friendly, container/Docker support Scalable from single machine to cluster or HPC environments Flow-based sequencing support Hardware acceleration (e.g., GPU/DRAGEN) Support for large sample-cohort variant calling CNV and methylation tools added	[26,32,36,39]

## 4. Scalability and Computational Efficiency

Scalability in GATK is largely achieved by parallelizing the data-partitioning process [20]. Throughout the early (GATK1–GATK3) releases, parallelization was facilitated through a MapReduce-based framework. In this framework, the input data is partitioned into independent units, allowing simultaneous processing by the same tool, depending on available CPU cores [20] (Figure 1). While MapReduce was not developed by the GATK authors, it was designed to efficiently handle large datasets on clusters of machines, from data processing to data generation, and became increasingly relevant as sequencing data volumes increased [22].

In simple terms, MapReduce uses two core functions, a *map* function that splits the input into multiple intermediate sets based on key–value pairs, and processes them independently, followed by a *reduce* function that aggregates the results into a single output [20,22] (Figure 1). Beyond time-saving process parallelization, MapReduce contributed to GATK’s ease of use [20,22]. Even users lacking experience with parallel and distributed systems could use MapReduce-based programs, as the framework automatically partitions input data, schedules execution, and manages process failures and inter-process communication [22].

Early versions of GATK used the MapReduce framework to structure data distribution and result aggregation within a multi-threaded architecture on single machines [20]. This approach addressed several challenges that affected early genomic analyses and variant callers, including execution speed, scalability, and ease of use [20]. However, performance bottlenecks related to memory management and intermediate file writing remained, motivating the adoption of new parallel processing frameworks to further optimize large-scale analyses [20,40].

GATK4 introduced support for Apache Spark, enabling distributed in-memory processing across clusters of machines to scale NGS analyses, particularly the variant calling, beyond single-node multithreading [30,36]. This architectural change allowed throughput to scale with available hardware resources, as well as improved handling of large datasets [30]. In parallel, improvements to the implementation (coding) of individual tools reduced computational overhead and further increased overall throughput [20]. Early GATK4 self-benchmarking studies reported up to an order of magnitude faster processing by HaplotypeCaller [36] and BQSR [37] compared to their earlier versions, while MarkDuplicates showed a three-fold increase in processing speed [37] (Table 1).

## 5. Reproducibility and Workflow Management

From its earliest releases to the current version, GATK has been designed as a platform-independent toolkit [20,36]. In parallel with internal architectural development, its execution model evolved to support diverse computing environments, enabling analyses to be performed on single machines, high-performance computing (HPC) clusters, cloud platforms, and hardware-accelerated systems [41,42,43]. In its early development stages, to support reproducibility across diverse environments, GATK utilized Queue, an internal command-line workflow and job management system that wrapped GATK commands into executable scripts for local and cluster-based environments [44]. While Queue facilitated structured pipeline execution, achieving reproducibility across different computing platforms motivated a shift toward standardized, machine-readable workflow descriptions [44].

Since the GATK3 era, GATK’s developer, the Broad Institute, introduced the now-recommended Workflow Description Language (WDL), together with the open-source execution engine Cromwell, for defining and executing GATK workflows [41,43]. WDL provides a standardized workflow description syntax for describing analytical steps, inputs, and outputs independently of the computing environment [41]. In turn, Cromwell interprets these workflow descriptions and schedules task execution across local machines, clusters, or cloud platforms, enabling consistent and reproducible analyses across users and infrastructures [41,42].

To further support reproducibility and accessibility, the Broad Institute maintains a public GitHub repository (https://github.com/broadinstitute/gatk; accessed on 30 March 2026) that contains curated WDL implementations of GATK Best Practices workflows [41]. These workflows cover common NGS data processing and variant calling tasks, have been tested by the developers, and are shared with the community as standardized, reusable analysis templates, thereby reducing user-to-user variability and supporting automated, repeatable execution.

Platforms such as Terra (formerly FireCloud) integrate Cromwell execution, additionally providing a graphical user interface (GUI) that facilitates workflow sharing, execution, and collaboration, particularly for users without extensive programming experience, while preserving reproducibility across local machines (laptops), clusters, and cloud providers [42]. Terra and Cromwell have been widely adopted by large-scale, Broad-led projects, including gnomAD [45].

In parallel to WDL-based solutions, community-developed workflow frameworks such as the Common Workflow Language (CWL) and Nextflow have emerged to promote portable and reproducible pipeline execution in a vendor-neutral manner [46,47]. Nextflow, in particular, has seen broad adoption in both academic and industrial bioinformatics and is used by large initiatives such as the Personal Genome Project UK [48]. The Nextflow ecosystem is further supported by the nf-core project [49], which provides peer-reviewed and ready-to-use GATK Best Practices workflows [50]. Together, WDL-Cromwell and CWL/Nextflow-based approaches still coexist in the GATK ecosystem, with WDL often favored for large-scale cloud-based projects and Nextflow providing flexibility and broader portability across a wide range of computing environments [47,50].

## 6. GATK’s Functional Scope

Through continuous refinement, GATK has expanded its functional scope into a broad ecosystem of command-line utilities (Figure S1), comprising 430 tools organized into 21 function-based categories in recent GATK4 releases [21,26] (Table 2). At the core of its functionality remains read pre-processing, followed by detection and genotyping of single-nucleotide variants (SNVs) and indels in diploid DNA, primarily performed using HaplotypeCaller for germline and Mutect2 for somatic variants [20,21,30]. However, particularly with the expansion of GATK4, additional variant classes such as CNV and structural variants (SVs) [21,33,51] are also supported, although to a lesser extent with comparatively narrower toolsets [21,26] (Figure 2).

**Figure 3:**
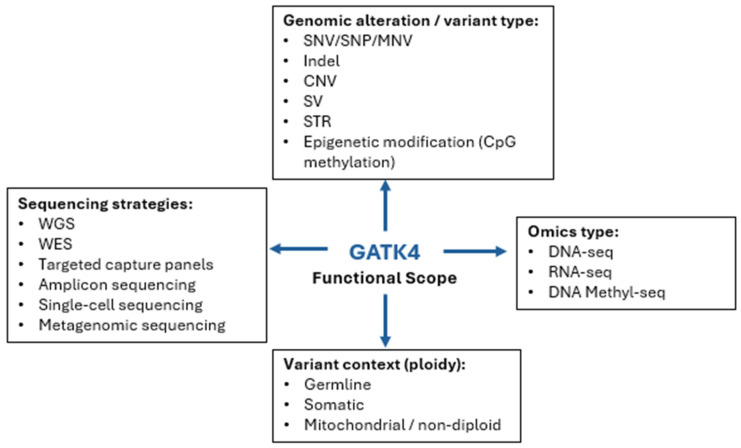
GATK’s current functional scope. The figure summarizes representative categories of genomic alteration types, sequencing strategies, omics modalities, and variant contexts to which GATK tools can be applied.

Although GATK supports multiple omics data types (Figure 3), methylation analyses are currently limited and centered around the experimental tool MethylationTypeCaller [21] (Table 2). RNA-seq analyses are largely conducted using the same core variant calling tools as DNA-seq data through parameter adaptation and workflow modifications [52]. Therefore, RNA-specific analyses do not constitute a separate functional category in the GATK tool index (Figure S1) [21,28].

The toolkit supports diverse sequencing strategies, including whole-genome sequencing (WGS), whole-exome sequencing (WES), targeted capture panels, amplicon sequencing, and single-cell sequencing through adapted Best Practices workflows [21] (Figure 3 and Table S1). As sequencing technologies evolve, GATK has incorporated compatibility with emerging platforms, including Ultima Genomics (since GATK4.3), and expanded support for additional data types such as methylation (since GATK4.0) and CNVs (since GATK4.0) (Table 2). Through this progressive expansion, GATK has evolved from an early SNP-centric germline variant discovery framework into a broad analytical ecosystem encompassing somatic oncology, cohort-scale joint genotyping, structural variant and CNV analysis, RNA-seq processing, and non-diploid variant models in GATK4 [14,20,21,30,33,51].

## 7. Variant Callers Before the GATK

GATK was not the first attempt at genotyping; several research groups had proposed alternative methods earlier [20,53,54]. Early pioneers emerged in the late 1990s with PolyBayes [55,56] and Phrap/PolyPhred in early genome resequencing-based projects [57,58], followed by GATK’s near-contemporaries MAQ [59], SOAPsnp [60], and Atlas-SNP2 [61]. Notably, these earlier methods largely relied on probabilistic frameworks, particularly Bayesian or likelihood-based models—the same approaches later adopted and further developed in GATK’s own tools, such as UnifiedGenotyper [62].

More specifically, most early germline short variant callers share a site-wise genotype framework in which evidence from a pileup of reads was converted into genotype likelihoods using base error probabilities, and then combined with a prior to yield posterior genotype probabilities and Phred-scaled confidence scores [16,59,63,64]. A compact formula (shown below) for Bayesian posterior inference over genotypes, with readwise likelihood factors, captures this shared core [16,59,63].

$$P\left(g|R\right)=\frac{P\left(g\right)\prod_{i=1}^{n}P\left(r_{i}|g\right)}{\sum_{g^{\prime }}P\left(g^{\prime }\right)\prod_{i=1}^{n}P\left(r_{i}|g^{\prime }\right)}$$

Here, *P*(*g*) is a prior on genotypes, and *P*(*r_i_*|*g*) is the per-read likelihood. In the simplest pileup model, *P*(*r_i_*|*g*) is derived from a Phred base quality score *Q_i_* interpreted as an error rate *ε_i_* = 10^−*Q_i_*/10^ and a diploid mixture model in which a read is assumed equally likely to originate from either allele.

The same mathematical structure appeared across early callers and was subject to several important limitations. First, the likelihood typically treated reads and their errors as conditionally independent and quality scores as calibrated error probabilities, such that any context dependence or miscalibration was magnified by the multiplicative aggregation of per-read likelihoods in the Bayesian posterior equation [16,59,63,64]. Second, the model assumed that the pileup alignment is correct [16,20,59,63]. Together, these assumptions limited the ability of early variant callers to account for several well-documented error modes:

### 7.1. Base-Quality Miscalibration

In SOAPsnp (https://github.com/zzhangjii/soapsnp; accessed on 30 March 2026) analyses of Illumina data, base quality scores were shown to be strongly miscalibrated in a substitution-class-dependent manner. For some substitution types, the discrepancy between reported and empirical error probabilities reached 58–72%. Moreover, for bases assigned a nominal Q10 score, observed substitution rates ranged from approximately 1.6% to 5.3%, rather than the expected 10% [16,60].

### 7.2. Duplicated Reads

In early large-scale low-coverage human WGS datasets such as the 1000 Genomes Project Pilot, approximately 20% of aligned reads were identified as duplicates using tools such as Picard (https://github.com/broadinstitute/picard; accessed on 30 March 2026) or SAMtools (https://github.com/samtools/samtools; accessed on 30 March 2026). Such duplicate alignments predominantly reflected PCR amplification artifacts and had important implications for downstream variant calling. Omitting duplicate marking, on the other hand, inflated support for heterozygous SNPs and indels [15,16,60].

### 7.3. Mapping Ambiguity

Highly repetitive, highly homologous, and structurally complex regions often yield ambiguously placed reads, leading to multi-mapping or mis-mapping [65], as exemplified by the pseudoautosomal regions shared between chromosomes X and Y [66]. In chromosome X analyses using SOAPsnp (https://github.com/zzhangjii/soapsnp; accessed on 30 March 2026), only approximately 88% of the chromosome was reported as covered by uniquely mappable reads, with the remaining regions described as highly repetitive and lacking confident unique alignments [59,60]. Such mapping ambiguity violates the assumption of correct read placement in pileup-based models and can inflate false-positive variant calls, distort allele balance, or render regions effectively inaccessible to reliable genotyping [16,67].

### 7.4. Reference Bias

Reference bias is described as a systematic shift toward reference allele support in short-read datasets. This arises because reads matching the reference sequence often align more readily or receive higher mapping confidence than reads carrying alternative alleles [16]. Such bias distorts allele balance and can influence genotype likelihood estimation in pileup-based callers [16,68].

### 7.5. Indel-Adjacent SNP Artifacts

Misalignment of reads spanning indels is a well-documented source of false SNP calls in early pileup-based callers. MAQ (https://maq.sourceforge.net/; accessed on 30 March 2026) demonstrated that incorporating mapping-quality weighting reduced false-positive SNPs, with one evaluation showing a reduction from 217 to 186 false calls. In the GATK framework, more than 15% of reads spanning known homozygous indels were reported as misaligned; local realignment corrected approximately 6.6 million reads and eliminated approximately 1.8 million mismatch loci [16,18,59,69].

### 7.6. Strand Bias, Read-Position Bias, and Cycle Bias

Early short-read datasets exhibited systematic error patterns in which sequencing error rates varied with sequencing machine cycle, read position, and strand orientation [16]. Strand bias refers to uneven support for an allele between forward and reverse reads and is used as an indicator of potential false-positive calls in standard GATK-era filtering [14]. Read-position bias arises when the alternate allele is observed preferentially near the ends of reads, which is likewise treated as indicative of error in pileup-based filtering [14]. Cycle-dependent error patterns motivated recalibration approaches in early callers, including SOAPsnp (https://github.com/zzhangjii/soapsnp; accessed on 30 March 2026) quality score recalibration and the related covariate-based recalibration implemented in GATK (including machine cycle and sequence context/dinucleotide context) [18,60,64,69].

### 7.7. Depth Extremes and Coverage Heterogeneity

Early pileup-based variant calling was highly sensitive to local sequencing depth [16,60,70,71,72]. In low-coverage WGS (~4×), insufficient read depth directly limited sensitivity, resulting in elevated false-negative rates. In WES and targeted sequencing, where local read depth can vary by more than an order of magnitude across regions, uneven coverage similarly reduces effective power to detect true non-reference sites [73]. Modeling studies have shown that achieving at least 13× local depth is required to detect heterozygous SNVs with high confidence; even at mean on-target depths around ~20×, 5–15% of heterozygous variants may remain undetected because local depth remains suboptimal [73,74].

To reduce false-positive SNPs, variant confirmation strategies based on orthogonal platforms (e.g., genotyping arrays, Sanger resequencing, or family-based inheritance constraints) were used to estimate concordance and error rates under known genotypes [56,60,75]. Callset-wide sanity checks treated the callset as a population-level object and evaluated whether global summary statistics were plausible, including the transition/transversion ratio (particularly among novel SNPs) [15,16,18,69,76]. Pileup-signature diagnostics interrogated the internal structure of read evidence, including strand imbalance tests, cycle- and read-position bias, allele-balance distributions at heterozygous sites, mapping-quality distributions, and near-indel clustering. These features can reveal alignment uncertainty, correlated reads, and reference or mapping bias that a site-wise caller might otherwise interpret as genuine support [16,18,59,60,68].

Notably, pre-HaplotypeCaller GATK pipelines included dedicated tools such as RealignerTargetCreator and IndelRealigner that collectively addressed base mismatches around indels commonly misinterpreted as clusters of SNPs [14]. These changes, however, did not alter the underlying genotype likelihood formulation, but instead modified the alignment input to that equation [14,16]. In comparison, HaplotypeCaller marked a major algorithmic shift in variant discovery, moving away from rather simplistic site-wise genotype likelihood evaluation directly from aligned reads toward assembling candidate haplotypes locally [30,77]. In such a model, genotype likelihoods are computed by assembling candidate haplotypes and then aligning reads to these haplotypes [77]. This shift can be expressed by the equation shown below [30,77].

$$P\left(g|R\right)\propto P\left(g\right)\prod_{i=1}^{n}\left(\sum_{h\;\in \;H\left(g\right)}P\left(r_{i}|h\right)P\left(h|g\right)\right)$$

Here, *g* is a candidate diploid genotype at a locus (e.g., AA, AB, BB), and *R* is the set of all sequencing reads overlapping the locus. *P*(*g*|*R*) is the posterior probability of genotype *g* given all reads. *P*(*g*) is the prior probability of genotype *g.* $\prod_{i\;=\;1}^{n}\;$ denotes a product over all reads. *h* ∈ *H*(*g*) is the set of candidate haplotypes consistent with genotype *g*. *P*(*r_i_*|*h*) is the likelihood of read *r_i_* given haplotype *h*, computed using a Pair Hidden Markov Model (Pair-HMM). *P*(*h*|*g*) is the probability that haplotype *h* is present under genotype *g*.

In summary, while the earlier variant callers advanced the field significantly, they often exhibited reduced sensitivity at low sequencing coverage [59], had comparatively limited error-modeling and filtering strategies [72], and showed relatively poor concordance across different tools [72].

## 8. GATK’s Best Practices

**Figure 4:**
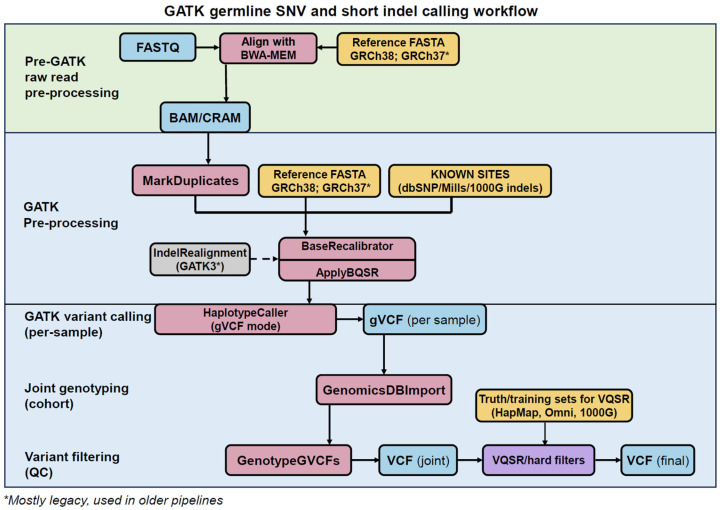
GATK Best Practices workflow for germline SNV and short indel discovery. Background shading indicates whether a process is handled within the GATK toolkit: green, pre-processing using tools external to GATK; blue, processes performed within GATK. Box colors indicate: blue, file formats; pink, native GATK functions; yellow, external downloadable resources utilized in GATK’s Best Practices. The germline variant calling workflow emphasizes three stages: (1) Pre-GATK raw read pre-processing, (2) GATK internal pre-processing, and (3) GATK variant calling.

While GATK is often retrospectively associated with its early scalable engine, its durable community impact is equally rooted in the formalization of methodological standards. From its inception, GATK operated on aligned reads in the Sequence Alignment/Map (SAM) format and its binary BAM representation [63], a standard alignment format introduced to unify and standardize aligner output across sequencing platforms and downstream tools [63] (Figure 4). This enabled GATK to support data generated from multiple sequencing platforms, including Illumina, SOLiD, 454 Life Sciences [18], and Complete Genomics [20], and thereby enabling interoperability across platforms and compute environments. Moreover, GATK was designed to analyze coordinate-sorted BAM files following alignment and standard pre-processing, and to remain compatible with most widely used short-read aligners [20].

In germline workflows, pre-processing steps such as duplicate marking (MarkDuplicates) and base quality score recalibration (BaseRecalibrator and ApplyBQSR; together BQSR) precede variant calling (Figure 4). Variant discovery is then performed using tools such as HaplotypeCaller (Figure 4), which evaluates evidence from aligned reads and locally assembled haplotypes against a reference genome to infer sample genotypes [14,16,77]. In typical cohort workflows, HaplotypeCaller produces an intermediate genomic Variant Call Format (gVCF) output, which is a standardized multi-column text file format used to store information about detected genetic variations [77,78]. Subsequently, gVCF undergoes joint genotyping and variant filtering to generate the final VCF output that can be used for clinical applications and genomic analyses [14] (Figure 4).

The “GATK Best Practices” reframed short-read variant calling from a collection of powerful but loosely coupled tools into a versioned, end-to-end reference workflow with recommended defaults, reference resources, and implementation guidance, thereby reducing methodological ambiguity and narrowing study-to-study methodological variability in germline calling [79]. This formalization crystallized between late 2013 and 2014 (Figure 2). Accordingly, formal Best Practices recommendations for RNA-seq variant calling were also introduced during this period, specifically, in the GATK3 era [14,16] (Figure 2). The Best Practices pipelines provided an explicit reads-to-variants workflow synthesized from large-scale project experience (including the 1000 Genomes Project), establishing a shared default for pre-processing, variant calling, and filtering decisions [14,80]. Central to this standardization were BQSR and probabilistic variant quality score recalibration (VQSR), which replaced heuristic hard filtering in large-scale germline workflows with model-based calibration of sequencing and variant-level error [14,16].

In parallel, the GATK team publicly articulated a “reference model” for incremental joint discovery—calling each sample in gVCF mode, followed by cohort genotyping—to solve the practical “N + 1” problem of growing cohorts while retaining site-level confidence information for joint inference [30,34,81]. This gVCF-based cohort strategy, now described as the recommended approach for GATK versions 3.0 and above, became the backbone of germline calling Best Practices and was disseminated as an explicit, reusable workflow rather than an ad hoc set of commands [82]. Moreover, to this day, GATK Best Practices workflows are designed to maximize sensitivity at the calling stage, followed by model-based filtration steps to remove biased or spurious variants [14].

Best Practices also evolved as a living standard maintained alongside the toolkit. As haplotype-based callers matured, GATK documentation updated recommended workflows to omit legacy indel realignment for HaplotypeCaller- and Mutect2-based pipelines, reflecting the algorithmic shift to local assembly/realignment within the caller [83]. The current cohort-calling guidance continues to position the gVCF joint-genotyping model as the recommended workflow designed to replace earlier multi-sample joint discovery approaches that become computationally unwieldy at scale [82].

Importantly, Best Practices are not only prescriptive documentation; they were adopted as a validation and production baseline in settings where end-to-end reproducibility mattered. Examples of GATK Best Practices implementation include the clinical WES/WGS pipeline by Linderman et al., 2014, which incorporates canonical pre-processing steps (e.g., duplicate marking, indel realignment, and BQSR) that could be validated as a single workflow unit [84], and the Alzheimer’s Disease Sequencing Project’s VCPA pipeline that performs cohort-level joint variant calling using GATK Best Practices [85]. Comparative variant calling performance analyses worldwide also treat the GATK Best Practices as the relevant benchmark target—for example, Heldenbrand et al., 2019, frame their runtime benchmarking from duplicate marking through variant calling explicitly around the Best Practices workflow (Table S1), underscoring its role as the community default configuration to optimize and deploy variant calling [39].

## 9. GATK’s Governance and Funding Model

GATK was developed at the Broad Institute, a nonprofit research institute jointly governed by MIT and Harvard University [16,20]. From the outset, GATK was created as a community resource rather than a commercial product, a choice that shaped its governance model and introduced sustainability challenges largely independent of its core algorithmic development [86].

Early GATK development was supported primarily by research grants from the U.S. National Human Genome Research Institute (NHGRI) awarded to Broad Institute investigators via NGS analyses in large-scale human genomics projects, most notably the 1000 Genomes Project (‘Joint SNP and CNV calling’ U01 HG005208 and ‘Large Scale Sequencing and Analysis of Genomes grant’ U54 HG003067) [16,20]. This grant-funded academic model enabled rapid methodological innovation and broad dissemination but did not ensure long-term resources for software development, maintenance, or user support. During the period spanning GATK1 and early GATK2 development, the toolkit was distributed under a fully open-source MIT License (Figure 2) [16,20].

As the GATK user base expanded beyond the Broad Institute, reportedly reaching approximately 100,000 users a month, sustaining active development became financially challenging under a purely grant-funded academic model [87]. In response, the Broad Institute introduced a dual-licensing model during the GATK2 and GATK3 era. Under this model, GATK remained freely available for academic and nonprofit research use while explicitly prohibiting for-profit use without a commercial license (Figure 2). The revenue from commercial licenses, administered through Appistry, helped support continued codebase maintenance, bug fixing, and expansion of user support [87,88]. During this period, commercial and non-commercial versions of GATK were distributed through separate GitHub repositories.

In 2015, the intermediary licensing arrangement with Appistry ended, and commercial licensing and support were transitioned directly to the Broad Institute [88]. This change simplified software distribution and aligned commercial releases more closely with current recommendations, including GATK Best Practices, as well as support for GATK and MuTect users [88]. However, the first major release of GATK4 transitioned the toolkit to a fully open-source model under the BSD license, fully eliminating commercial licensing fees and allowing unrestricted academic and commercial use [89]. GATK4.2 adopted a functionally equivalent license, Apache 2.0, that only differs by including a patent license-granting clause regarding practical reuse of open-source software [90].

This licensing transition coincided with major industry partnerships, including collaborations with Illumina, Intel, Microsoft, and cloud service providers [91], as well as three competitive NHGRI grants awarded to the Broad Institute for genomic technology development and large-scale variation analysis. The financial implications of these partnerships and grants for GATK development have not been discussed in the public domain. GATK’s governance within a well-resourced institutional framework such as the Broad Institute, combined with diversified funding sources, appears to provide a more durable sustainability model than reliance on short-term research grants alone.

## 10. Contributions to Science

Arguably, the contribution of the GATK to modern genomics has been profound. Without a widely accepted and rigorously maintained standard such as GATK, laboratories and consortia worldwide would likely have developed independent, incompatible pipelines for variant calling, hindering reproducibility, benchmarking, and cross-study comparisons. The existence of a common toolkit allowed consistent evaluation of variant calling accuracy across studies and stimulated the development of Best Practices workflows and pipelines that have continuously evolved with sequencing technologies, reaching high levels of sophistication in sensitive areas such as systematic error handling.

Software for genomics is a critical part of research infrastructure, funded by millions to tens of millions of dollars every year by major genome projects, NGOs, governments, and industrial entities [92]. If expressed in economic terms, the cumulative R&D cost savings from shared software infrastructure and methodological standardization such as GATK arguably reach millions of dollars over the past decade and will likely continue to grow as sequencing scales globally. For about 15 years, GATK has been central to most large-scale genomics projects that include variant calling (Table 3) [93], from population-scale efforts such as the 1000 Genomes Project [24] and gnomAD [45], to cancer genomics [20,25], small-scale personal genome projects [94,95,96], and disease association [97,98] or case studies [99,100,101]. The toolkit has also both directly and indirectly facilitated the generation of population reference panels, enabling genotype imputation in studies lacking matched controls (e.g., somatic mutation analysis in leukemia) [102] and salvaging samples with high genotype missingness, as in DNA extracted from degraded/contaminated tissues or ancient bones, thus contributing to forensics and anthropology. Furthermore, GATK has become a de facto standard for benchmarking sequencing platforms and variant calling algorithms [103]. In parallel, DRAGEN-GATK has contributed to uncovering rare and novel pathogenic variants in human genetic disorders [104,105,106,107,108].

As of 19 November 2025, the GATK suite’s four core publications (McKenna et al., 2010 [20]; DePristo et al., 2011 [16]; Van der Auwera et al., 2013 [14]; Poplin et al., 2017 [30]) collectively accumulated over 36,346 citations in Scopus and bioRxiv, and likely more projects used it than formally cited it. GATK papers collectively rank among the most cited in bioinformatics, comparable to other staple tools such as UCSC Genome Browser, FASTA, and HMMer [92].

**Table 3:** Summary of large-scale genome projects (≥1000 participants) and their use of GATK. Asterisk (*) denotes supplementary use limited to performance benchmarking.

Project	Number of Participants	Target Population	GATK Used
All of Us Research Program	>1,000,000	General population and patients (resident of the USA older than 18)	Yes [109,110]
Million Veteran Program	>800,000	U.S. veterans	No, genotyping arrays were used [111]
China Kadoorie Biobank	~512,000	Chinese adult population	No, genotyping arrays were used [112]
UK Biobank WGS/array project	>500,000	UK general adult population	Yes [113]
FinnGen	500,000	Not disclosed	Yes [114]
TOPMed (Trans-Omics for Precision Medicine)	>200,000	General U.S. diverse population + disease cohorts	No, ”GotCloud” pipeline was used [115]
Genomics Health Futures Mission (Australia)	>200,000	Target population depends on the sub-project (Various diseases and controls)	Yes [116,117]
Biobank Japan	~200,000	Japanese disease cohorts & controls	No, genotyping arrays were used [118]
gnomAD (Genome Aggregation Database)	~150,000	Global population reference	Yes [45]
UCLA ATLAS Community Health Initiative	150,000	Individuals from Los Angeles area under UCLA Health system	No, genotyping arrays were used [119]
100,000 Genome Project	100,000	Patients affected by a rare disease or cancer (85,000), their family members	Yes [18,19,23,24]
Saudi Genome Program	100,000	Saudi nationals	Not disclosed [120]
Turkish Genome Project	100,000	Healthy general population, rare disease patients, complex disease patients	Not disclosed [121]
Estonian Biobank Project	>52,000	General Estonian population (Adults)	Yes [122,123]
Genome Canada’s National Precision Medicine Initiative	30,000	10,000 Rare disease trios	Not disclosed [124]
International Cancer Genome Consortium (ICGC)	>25,000	Patients with cancer	Yes [125]
The Cancer Genome Atlas (TCGA)	~11,000	Patients with cancer	Yes [25]
The ChinaMAP	10,588	Noncommunicable Disease cohort 150,000 participants; Chinese diabetic Individuals 250,000 participants; Cardiovascular risk cohort ~450,000 participants	Yes [126]
Qatar Genome Programme	>10,000	Qatari general population	Yes [127,128]
UK10K	10,000	Patients with various health conditions (6000), General population (4000)	Yes [93,129]
Genomics Thailand Project	10,000	Patients with cancer	Not disclosed [130]
The GenomeIndia Project	10,000	Healthy and unrelated ethnic Indians from 83 populations	Not disclosed [131]
The Genome Russia project	3000	General Russian population (Geographically spread sampling)	Yes * (for benchmarking) [132,133]
deCODE	2636	General Icelandic population (All)	Yes [134]
Korea1k	1094	General Korean population (All)	Yes [102]
1KJPN	1070	General Japanese population (Healthy)	Yes [135]
Welfare Genomics Project (WGP)	1000	General Korean population (All)	Yes [136]
SweGen	1000	General population (942 participants were 1 of the twins)	Yes [137]
1000 Arab genome project	1000	Healthy and diseased individuals (individuals with citizenship)	Yes [138,139]

## 11. Current Limitations of GATK

GATK’s methodological design and Best Practices were developed primarily for human Illumina sequencing data [79]; therefore, its statistical assumptions limit performance outside this domain in several ways (Table 4). For instance, default GATK settings often implicitly assume human levels of heterozygosity and the availability of well-curated variant filtering resources (Figure 4), which restricts direct methodological transfer to many non-human genomes [16]. Resource availability directly affects VQSR, as it relies on large, high-confidence truth sets such as HapMap [140] and the 1000 Genomes Project SNP and indel sets [141]. In their absence, VQSR may become inapplicable or unstable, requiring hard filtering or the generation of organism-specific resources [16].

Another consequence of human-centric GATK optimization is that many statistical components were developed and benchmarked on diploid genomes and human patterns of genetic variation [14,16] (Table 4). In polyploid plants or mixed microbial populations, allele dosage estimation and variant representation become substantially more complex, and specialized callers such as LoFreq [142] may perform better [143,144]. For instance, viral quasispecies analyses often require confident variant detection at very low allele fractions. Consistent with this limitation, HaplotypeCaller explicitly cautions that it is “not well suited to extreme allele frequencies relative to ploidy” [77], while Mutect2 was developed within a tumor–normal variant calling framework, and its documentation does not describe explicit modeling of within-sample haplotype diversity typical of viral or microbial quasispecies [35].

**Table 4:** Summary of GATK limitations and points for improvement.

Design Bias	Root Cause	Practical Implications
Human-centric [14,16,20,141]	Many statistical assumptions were developed using human population data. Default resources (HapMap, Omni, 1000 G, Mills indels) are human-specific	Performance outside human genomes may require disabling VQSR, retuning parameters, or using hard filtering, potentially reducing accuracy
Short-read centric [16,145,146]	Core tools (BQSR, HaplotypeCaller, Mutect2) were developed and optimized for Illumina-style short reads (~100–250 bp) with SNP-dominant error profiles	Long-read platforms (ONT, PacBio) exhibit different systematic errors (indels, homopolymers), where specialized long-read callers often outperform GATK
Diploid-centric [16,143,144]	Default ploidy = 2. Genotype likelihood models scale poorly with high ploidy. Somatic models assume diploid baseline	Polyploid species (e.g., plants), highly aneuploid tumors, or mosaic samples may require careful parameter tuning and increased computation and run times
DNA-centric [14,147,148]	GATK was primarily designed for germline and somatic DNA variant calling. RNA-seq support is secondary, requires parameter tuning	RNA-specific artifacts (allele-specific expression, splice junction bias) complicate variant interpretation
SV, CNV limitations [149,150]	Limited support for SVs and CNVs compared to specialized tools	Other SV callers required for comprehensive genome analysis
Dependence on high-quality linear reference genome [9,151,152]	GATK assumes a well-assembled linear reference genome and reliable mapping	Performance may decline in highly polymorphic regions, repetitive genomes, or species where graph references are more appropriate
Performance in structurally complex genomes [153,154]	Many non-mammalian genomes (plants, microbes) have high repeat content, variable ploidy, segmental duplications, or mixed populations	Requires parameter adjustment; alternative tools may be better suited for microbial population genomics or polyploids
VQSR constraints [14]	VQSR requires large cohorts and high-confidence truth sets	Case studies, small, rare disease cohorts, and non-model organisms often rely on hard filtering, reducing statistical calibration robustness
Computational and parameter complexity [21,51,155,156]	Large number of tunable parameters, syntax changes between versions, and multi-step workflows al increase complexity	Risk of misconfiguration, steep learning curve for users without bioinformatics background

In addition, GATK’s reliance on a linear reference genome constrains variant discovery in highly diverse or structurally complex genomes (Table 4). Graph-based approaches have demonstrated improved read mapping and variant representation in regions containing large insertions, inversions, or population-specific haplotypes, highlighting limitations of strictly linear-reference workflows [151,152]. Another implicit assumption is the availability of a high-quality reference genome that closely represents the studied population. BQSR excludes known polymorphic sites from its error model so that remaining mismatches to the reference are treated as sequencing errors [14,16]. This assumption may become unreliable in phylogenetic or highly divergent contexts, particularly when true divergence from the reference is dense, and well-curated known-site catalogs are unavailable [157].

GATK’s algorithms were optimized for short-read Illumina-like DNA sequencing data and substitution-dominated error profiles [16] (Table 4). Although RNA-seq variant calling workflows exist, RNA-derived variants are confounded by splicing, allele-specific expression, RNA editing, and coverage biases, requiring additional pre-processing [77,147,148]. Similarly, Ion Torrent data exhibit homopolymer-associated indel artifacts and elevated false-positive indel rates that deviate from Illumina-tuned error assumptions [158]; therefore, existing GATK pipelines and Best Practices must be adapted and parameters adjusted [159]. In addition, SV and CNV detection are supported in GATK but are outside GATK’s primary design focus [149,150]. While GATK4 introduced germline CNV calling, complex rearrangements and large insertions are often more comprehensively resolved using long-read sequencing and tools developed specifically for SV detection [145,160].

Collectively, these constraints indicate that GATK is optimized for diploid, short-read, human DNA sequencing contexts. Non-human, particularly non-mammalian, polyploid, structurally complex, or transcriptome- and methylome-derived datasets often require substantial parameter adjustments, complementary methods, or alternative variant calling frameworks (Table 4).

## 12. Current Performance and Future Outlook

Contemporary benchmarking initiatives such as Genome in a Bottle (GIAB) show that GATK’s HaplotypeCaller remains a strong and widely used baseline for germline variant discovery [161,162,163], outperforming older variant callers such as FreeBayes in standard truth set evaluations (NA12878), with particular advantage in indel calling performance irrespective of aligner choice [163]. However, it is notable that HaplotypeCaller’s predecessor, UnifiedGenotyper, was among the genotyping tools used to create the GIAB gold standard indel set, which may create biases in benchmarking [163]. Regardless, newer approaches—notably the machine-learning caller DeepVariant and software- or hardware-optimized methods such as Strelka2 or DRAGEN—are able to match or exceed HaplotypeCaller for efficiency and SNV and small-indel accuracy in certain GIAB truth set benchmarking contexts [162,164] (Table S1). However, relative performance depends strongly on genomic region and variant type [163,165]: callers can perform nearly identically inside high-confidence regions but diverge substantially in difficult-to-map or low-coverage regions [164]. HaplotypeCaller performance may also be limited in high-coverage WES datasets, affected by uneven coverage, particularly when neural network-based filtering methods are applied [164], or exhibit precision limitations typical of short-read-based technologies in low-complexity/low mappability regions and within SV regions [161,166]. These are notable challenges to overcome, leaving space for niche variant callers tuned for specific uses.

Beyond benchmarking considerations, GATK continues to adapt to an evolving landscape of sequencing platforms, data types, and analytical demands (Table 2). For example, the relatively recent short-read sequencing platform BGISEQ-500 can be easily incorporated into a standard BWA + GATK workflow [167] (Figure 4) by using functions such as AddOrReplaceReadGroups [21]. However, GATK was originally designed and optimized for high-accuracy short reads, with specific assumptions about read error profiles and mapping behavior [20]. As third-generation sequencing (TGS) platforms for long and ultra-long reads, such as Oxford Nanopore Technologies (ONT) and Pacific Biosciences (PacBio), emerge with variant calling accuracy approaching that of Illumina for SNV [168], they provide complementary advantages such as accurate SV discovery. Correspondingly, the TGS variant calling is evolving through parallel toolchains [168,169] (https://github.com/oicr-gsi/guppy; accessed on 19 November 2026), (https://github.com/nanoporetech/dorado/; accessed on 19 November 2026), (https://github.com/nanoporetech/bonito; accessed on 19 November 2026) rather than being fully integrated into GATK.

In parallel with the primarily CPU-based GATK 4 ecosystem (~2019), hardware-accelerated implementations have emerged to meet the increasing throughput demands of large-scale genomic projects and clinical sequencing. Illumina’s DRAGEN platform, focused on proprietary acceleration of genomic analyses [170], in collaboration with the Broad Institute’s open-source GATK, introduced hybrid DRAGEN-compatible GATK workflows (DRAGEN-GATK) [171] (Figure 2). These implementations preserve GATK-compatible outputs while substantially reducing runtimes (Table S1), thereby increasing throughput and reducing sample turnaround time without sacrificing accuracy [171,172]. Such developments point toward a future in which optimized hardware acceleration becomes an operational standard for large-scale and clinical variant calling while retaining the established GATK analytical framework.

Despite rapid advances in sequencing technologies and pressures to keep up with technical innovations, GATK is unlikely to face obsolescence in the near future. In theory, the improving accuracy of TGS, combined with small-scale hardware, relatively low-cost workflow, and the added benefit of long-read coverage enabling accurate detection of large structural variants and repetitive regions, could gradually replace NGS as a leading sequencing technology [168,169]. However, ‘hybrid’ approaches appear more realistic, utilizing high-throughput short-read sequencing for cost-effective bulk data at a single base resolution, while long-read sequencing resolves structural variation and copy number complexities [173,174]. Furthermore, GATK is already deeply entrenched as foundational ‘infrastructure’ across genomics, bioinformatics, and healthcare research [17,175,176]. As the new sequencing, variant calling, and analysis methods mature, GATK—and its optimized iterations such as DRAGEN—remain the benchmark standard [164,177].

## 13. Conclusions

GATK is a massive and widely adopted NGS analysis toolkit that has been around for more than a decade. It takes aligned sequencing reads as input and provides a complete suite of tools for producing high-quality VCF files, a common endpoint of data processing and a starting point for downstream analyses. GATK’s major contribution has been its role in standardizing NGS variant calling practices by offering a rigorously maintained and continually improved toolchain, as well as Best Practices workflow recommendations. GATK stands out for its sustained maintenance, active and innovative development, and well-curated suite of tools that support both mainstream and specialized NGS workflows at any scale (Table 2). Throughout its evolution, it has consistently focused on improving variant calling accuracy, speed, scale, and error handling. Over the past 15 years, GATK has contributed—often implicitly—to numerous small- and large-scale genomic studies, including population genetics initiatives (Table 3), cancer genome projects, the construction of population reference panels, and studies benchmarking variant calling performance using reference datasets. However, GATK remains primarily human-centric and optimized for short-read NGS, especially for DNA data (Table 4). This may become a significant limitation as demand for sequencing other species increases and interest in other types of omics rises globally. Notably, long-read TGS technologies continue to mature, improve in accuracy, and expand in adoption, all of which may result in either competitive or cooperative dynamics with short-read NGS, consequently affecting global demand. Nevertheless, even if the GATK toolkit is no longer at the center of variant calling innovation in the future, it will continue to function as the foundational benchmark for newer technologies instead.

## Figures and Tables

**Table 2:** Summary of GATK’s tool availability based on the scope of analyses. This table highlights GATK toolkit expansion. Functional categories absent in early versions are marked with ×.

Functional Category [26]	Representative Tools	Tools in GATK4.6 [26]	Tools in GATK3.7 [51]	Tools in GATK2.8 [33]
Copy Number Variant Discovery	GermlineCNVCaller	11	×	×
Coverage Analysis	CountBases, CountReads	10 + 5 beta	×	×
Diagnostics and Quality Control	CompareBaseQualities, CollectRrbsMetrics (Picard)	45 + 8 beta +2 experimental	31	33
Intervals Manipulation	IntervalListTools (Picard)	6	×	×
Metagenomics	PathSeqPipelineSpark, PathSeqScoreSpark	6	×	×
Read Data Manipulation	AddOrReplaceReadGroups (Picard), ApplyBQSR, BaseRecalibrator	42 + 10 beta +3 experimental	8	9
Reference	CreateSequenceDictionary (Picard)	11 + 2 beta +2 experimental	3	2
Short Variant Discovery	GenotypeGVCFs, HaplotypeCaller, Mutect2	8 + 3 beta +1 experimental	9	10
Structural Variant Discovery	SVAnnotate	1 + 10 beta +2 experimental	×	×
Variant Evaluation and Refinement	CountVariants, ValidateVariants	13 + 4 beta +2 experimental	20 across 2 categories	23 across 2 categories
Variant Filtering	ApplyVQSR, FilterVcf (Picard)	6 + 4 beta +1 experimental	×	×
Variant Manipulation	GatherVcfs (Picard), SortVcf (Picard)	18	16	×
Base Calling	ExtractIlluminaBarcodes (Picard), IlluminaBasecallsToFastq (Picard)	7	×	×
Flow Based Tools	GroundTruthReadsBuilder	5 experimental	×	×
Genotyping Arrays Manipulation	CombineGenotypingArrayVcfs (Picard)	7	×	×
Methylation-Specific Tools	MethylationTypeCaller	1 experimental	×	×
Metrics	AlignmentSummaryMetrics	56	×	×
Flow Annotations	GcContent	9	×	×
Read Filters	LibraryReadFilter, MappedReadFilter	55	30	27
Variant Annotations	BaseQuality, Coverage, AlleleFraction	44	53	32
Other	IndexFeatureFile	4 + 3 BETA +1 EXPERIMENTAL	8 across 2 categories	18 across 2 categories

## Data Availability

No new data were created or analyzed in this study. Data sharing is not applicable to this article.

## Supplementary Materials

The following supporting information can be downloaded at: https://www.mdpi.com/article/10.3390/ijms27093754/s1. Reference [178] is cited in the Supplementary Material.
