# Supplementary figures and images for: Fifteen Years of the Genome Analysis Toolkit as the De Facto Standard in Short-Read Variant Calling
Source: Int J Mol Sci. 2026 Apr 23;27(9):3754. doi: 10.3390/ijms27093754 (PMC13164212; doi:10.3390/ijms27093754)

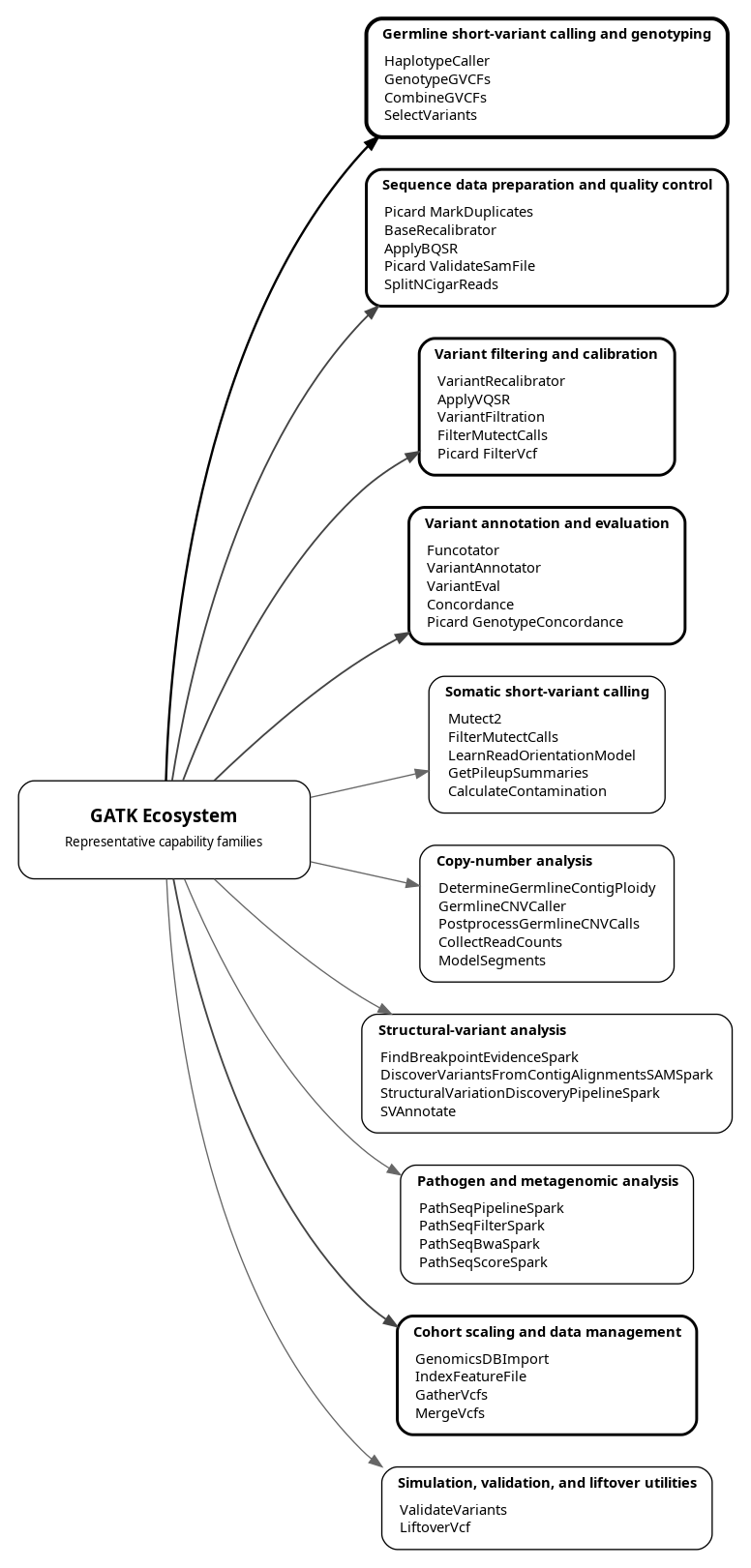

Supplement: Supplementary file 1 [file ijms-27-03754-s001.zip › GATK_analysis_ecosystem_FigureS1.png]
